# MAPK signaling pathways in host immune regulation during Mycobacterium tuberculosis infection

**DOI:** 10.3389/fimmu.2026.1781320

**Published:** 2026-03-18

**Authors:** Yi-fan Diao, Jia-yi Wang, Xue-ying Tan, Jiang-shui Yuan

**Affiliations:** 1 School of Medical Laboratory, Shandong Second Medical University, Weifang, China; 2 Clinical Laboratory Department, Qingdao Municipal Hospital, Qingdao, China

**Keywords:** anti-tuberculosis immunity, inflammatory signaling, MAPK signaling pathway, targeted therapy, tuberculosis

## Abstract

Tuberculosis (TB) is one of the world’s leading causes of death from a single infectious agent, with Mycobacterium tuberculosis (Mtb) primarily infecting the lungs via the respiratory tract. Following infection, immune cells such as macrophages and neutrophils phagocytose Mtb and initiate complex inflammatory and immune responses, driving the formation of granulomas and cavities within the lungs, ultimately leading to structural damage. In this intricate cascade, the MAPK signaling emerges as a critical regulator, orchestrating various cellular processes including inflammatory signaling, autophagy, apoptosis, and immune cell differentiation. Emerging evidence indicates that MAPK signaling critically shapes anti-TB immunity predominantly within macrophages, neutrophils, T cells and dendritic cells. Through extensive crosstalk among immune cells, MAPK signaling influences both host defense and disease progression. This review systematically summarizes current advances in understanding MAPK-mediated immune regulation during TB infection, with particular emphasis on the distinct roles of p38, ERK, and JNK signaling pathways. Furthermore, we discuss emerging therapeutic strategies to enhance anti-mycobacterial immunity by targeting MAPK signaling, thereby providing a valuable theoretical framework for the development of novel TB treatments.

## 1 Introduction

Tuberculosis (TB), caused by Mycobacterium tuberculosis (Mtb), is one of the chronic infectious diseases that poses a serious threat to public health (1, 2). More than 10 million people worldwide are diagnosed with TB, and 1.23 million patients die from TB in every year (3). The increasing prevalence of multidrug-resistant TB (MDR-TB) poses significant challenges for treatment and highlights the urgent need for novel therapeutic strategies beyond conventional antibiotics (4). Understanding the host immune responses that determine infection outcome is therefore critical for developing effective host-directed therapies (HDTs).

Mtb infection triggers a complex host immune response involving both innate and adaptive immunity (5). Mtb primarily infects the lungs via the respiratory tract, where it is phagocytosed by innate immune cells, including alveolar macrophages and neutrophils. These cells initiating inflammatory signaling cascades and recruit more innate and adaptive immune cells such as dendritic cells (DCs) and T lymphocytes. These cellular responses aim to restrict bacterial growth, activate antimicrobial mechanisms such as autophagy and apoptosis, and ultimately form granulomas that contain infection (6, 7). However, Mtb has evolved diverse strategies to manipulate host signaling networks and evade immune clearance, enabling long-term persistence and latent infection. Excessive or dysregulated inflammation can also lead to progressive lung tissue damage and impaired immune defense (8).

Mitogen-Activated Protein Kinase (MAPK) forms a conserved kinase cascade that transmits extracellular stress signals to the nucleus, including bacterial infections, oxidative stress, and cytokines, thereby regulates cellular response (9, 10). During TB infection, MAPK pathways regulate key immune processes, such as cytokine production, autophagy, apoptosis, and immune cell differentiation (11–15). Through extensive crosstalk among immune cell types, MAPK pathways coordinate the balance between host defense and immunopathology.

In this review, we summarize recent advances in the understanding of MAPK signaling in innate and adaptive immune cells during Mtb infection, analyze the mechanistic roles of p38, ERK, and JNK in immune regulation, and discuss current progress in developing MAPK-targeted therapeutic strategies. By highlighting the complexity and translational potential of MAPK-mediated regulation, this review aims to provide new perspectives for the development of effective HDT-based TB therapies.

## 2 MAPK signaling pathways: core components and regulatory features

The Mitogen-Activated Protein Kinase (MAPK) family is a conserved group of serine/threonine protein kinase (16), including p38 MAPK, extracellular signal-regulated kinase (ERK), and c-Jun N-terminal kinase (JNK) (17). MAPK signaling pathways function as highly conserved yet versatile signaling modules that enable immune cells to sense and integrate diverse environmental cues during Mtb infection. Canonically, MAPK signaling are composed of a hierarchical kinase cascade in which extracellular stimuli activate upstream MAPK kinase kinases (MAP3Ks), followed by sequential activation of MAPK kinases (MAP2Ks) and downstream MAPKs (18).

Activation of MAPK signaling is initiated by the recognition of mycobacterial components through pattern recognition receptors, including Toll-like receptors(TLR), C-type lectin receptors(CLR), and intracellular NOD-like receptors(NLR) (19, 20), which converge on key MAP3Ks such as TAK1, ASK1, and members of the MEKK family (10). These upstream kinases selectively activate distinct MAP2Ks, including MEK1/2, MKK4/7, and MKK3/6, thereby conferring pathway specificity to ERK, JNK, and p38 mediated signaling, respectively (11, 21, 22). Once activated, MAPKs phosphorylate a wide array of nuclear and cytoplasmic substrates, enabling immune cells to exert its biological effects (23).

**Figure 1:**
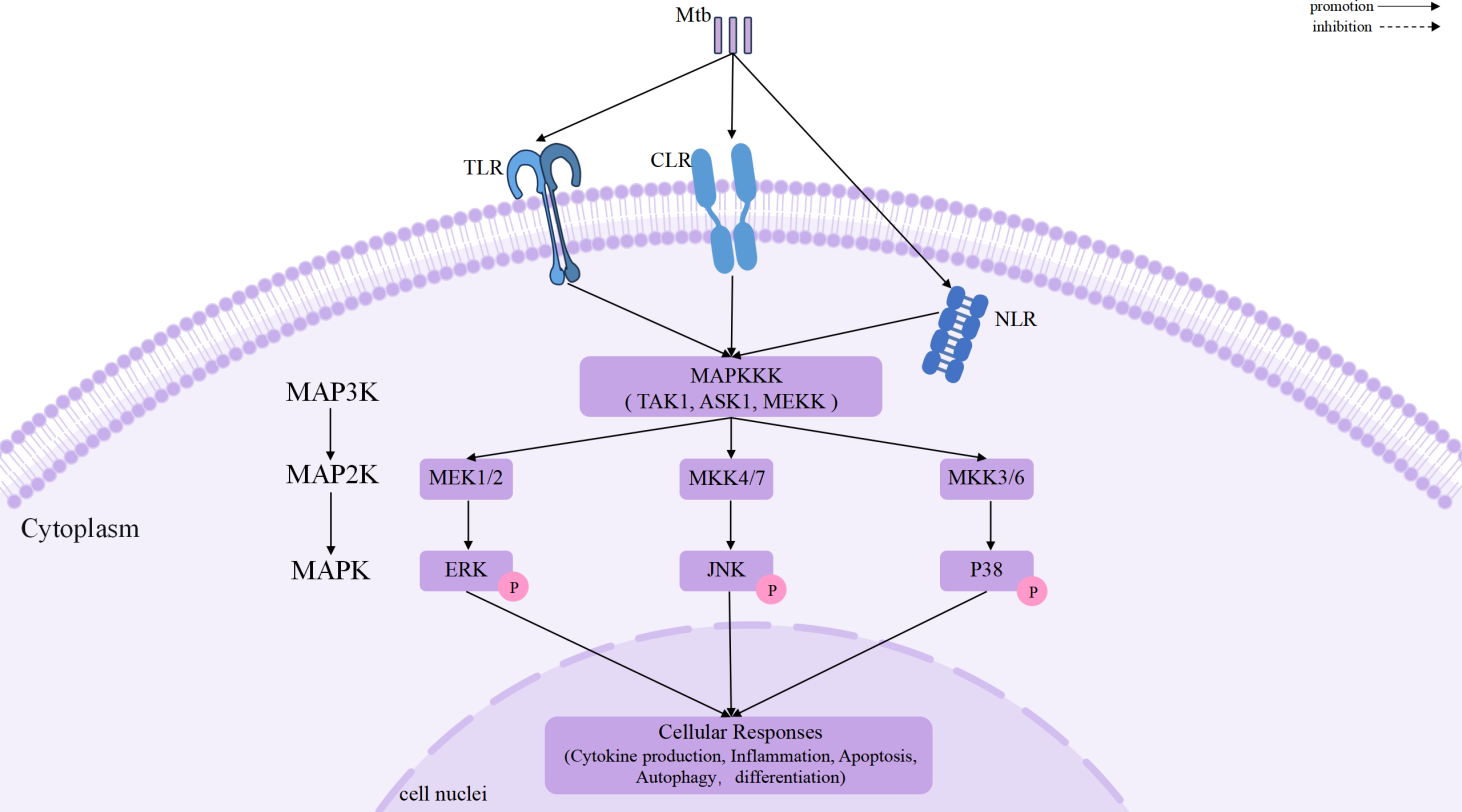
Canonical MAPK signaling cascades activated during Mtb infection.

Importantly, MAPK signaling is tightly regulated at multiple levels. Dual-specificity phosphatases (DUSPs), scaffold proteins, and feedback loops fine-tune signal amplitude, duration, and spatial distribution, conferring context-dependent outcomes (24, 25). Rather than functioning as simple on–off switches, MAPK pathways exhibit dynamic signaling behaviors, in which signal strength and temporal patterns critically determine whether immune responses favor antimicrobial defense or immunopathology (10). A schematic overview of the canonical MAPK signaling cascades and their regulatory components is shown in **Figure 1**.

## 3 Immune cell involvement in MAPK signaling during Mtb infection

### 3.1 Global activation of MAPK signaling across immune cell populations

Accumulating evidence indicates that MAPK signaling pathways are broadly activated across multiple immune cell populations during Mtb infection, including macrophages, neutrophils, DCs, and T lymphocytes (26, 27). This widespread activation reflects a conserved host response to mycobacterial challenge rather than a cell-type–restricted phenomenon. Upon infection, Mtb-derived components engage pattern recognition receptors (PRRs), such as Toll-like receptors and NOD-like receptors, leading to rapid activation of the p38, ERK, and JNK MAPK (28–30).

Across diverse immune compartments, MAPK signaling regulates a shared set of effector programs that are central to TB immunity. These include the induction of pro-inflammatory cytokines and chemokines, modulation of immune cell activation and polarization states, and regulation of stress-responsive cellular processes that influence host–pathogen interactions (31, 32). Importantly, MAPK activation often operates in concert with NF-κB and other transcriptional regulators, forming an integrated signaling network that amplifies or restrains inflammatory responses depending on the immunological context (27, 33).

Together, these observations establish MAPK pathways as ubiquitously engaged signaling axes in TB immunity. This global activation provides the mechanistic foundation upon which cell-type–specific functional biases and cross-cellular coordination emerge, as discussed in the following sections.

### 3.2 Functional biases of MAPK signaling in major immune cell types

Although MAPK signaling pathways are broadly activated across diverse immune cell populations during Mtb infection and universally contribute to inflammatory cytokine production, their immunological impact is shaped by cell-specific contexts (34). Rather than conferring exclusive signaling functions, MAPK pathways exhibit functional biases by prioritizing distinct immune programs within different cell types (35, 36). These biases reflect differences in cellular roles, downstream effector coupling, and temporal dynamics of MAPK activation during TB.

Macrophages are the primary regulators of the inflammatory response during infection and serve as the first line of defense against Mtb (37, 38). Accordingly, MAPK signaling in macrophages integrates inflammatory activation with intracellular antimicrobial mechanisms. The Mtb virulence-associated factor Yrb3EA activates the JNK and NF-κB signaling pathways, leading to increased expression of inflammatory cytokines such as tumor necrosis factor(TNF) and interleukin-6 (IL-6), thereby enhancing macrophage bactericidal activity, and inhibiting Mtb replication (39). In contrast, Mtb can also subvert MAPK signaling to promote intracellular survival. Mtb induces phosphorylation and nuclear translocation of cAMP Response Element Binding Protein(CREB) via p38 MAPK, suppresses NF-kB translocation, and upregulates the expression of specific CREB-regulated immediate early genes (IEGs), such as cyclooxygenase-2 (COX-2), MCL-1, CCL8, and c-FOS, which inhibits fusion of the Mtb-containing phagosome with lysosomes, preventing Mtb’s destruction (40).

Neutrophils rapidly accumulate at sites of Mtb infection, particularly in the lung, and contribute to early antimicrobial defense through phagocytosis and the formation of neutrophil extracellular traps (NETs) (41). During early infection, activation of MAPK pathways, including ERK, p38, and JNK, downstream of TLR2 and TLR4 promotes NF-κB signaling and induces the production of IL-6 and IL-23, enhancing neutrophil-mediated killing of Mtb (42). However, as infection progresses, sustained MAPK-driven activation in neutrophils can amplify cytokine and chemokine release, including IL-8, IL-13, and IL-6, leading to excessive inflammation and lung tissue damage (43). These observations suggest that, while MAPK signaling in neutrophils shares common inflammatory outputs with other immune cells, its functional bias lies in regulating the magnitude and duration of inflammation, thereby influencing the balance between early host protection and late-stage immunopathology (43, 44).

In contrast to innate immune cells, MAPK signaling in T lymphocytes primarily shapes adaptive immune responses (45). MAPK pathways are activated indirectly during TB through antigen presentation and cytokine signaling, contributing to T cell activation, expansion, and differentiation. For example, Mtb proteins such as Rv1876 and Rv2029c respectively activate MAPK and NF-κB signaling in DCs and macrophages, thereby enhancing antigen presentation, which in turn promotes T helper type 1 (Th1) responses, expansion of effector and memory T cell populations, and reduction of bacterial burden (46, 47). In this context, MAPK signaling in T cells is functionally biased toward regulating cell fate decisions, effector differentiation, and the establishment of long-term adaptive immunity (48).

DCs serve as critical intermediaries between innate sensing and adaptive immune activation, and MAPK signaling in DCs reflects this coordinating role (49, 50). Activation of MAPK pathways promote DCs maturation, costimulatory molecule expression, and cytokine production that instruct T cells polarization (51, 52). Mtb-derived proteins such as Rv3628 activate ERK, p38, and JNK signaling through TLR- and MyD88-dependent pathways, leading to DCs activation, polarization of Th1 phenotype and expansion of memory T cells (53). Through these mechanisms, MAPK signaling in DCs is functionally biased toward shaping antigen presentation and directing downstream adaptive immune responses.

Taken together, MAPK signaling during Mtb infection displays functional convergence at the level of inflammatory activation across immune cell types, while exhibiting context-dependent biases in downstream immune programs. In macrophages, MAPK pathways preferentially integrate inflammation with intracellular bacterial control; in neutrophils, they modulate inflammatory amplification and tissue injury; in T cells, they govern differentiation and memory formation; and in dendritic cells, they coordinate antigen presentation and immune polarization. This functional bias framework provides a coherent conceptual basis for understanding how MAPK signaling orchestrates multi-cellular immune responses during TB and sets the stage for subsequent analysis of shared MAPK-regulated functional outcomes.

## 4 MAPK-mediated immunological functions in tuberculosis

**Figure 2:**
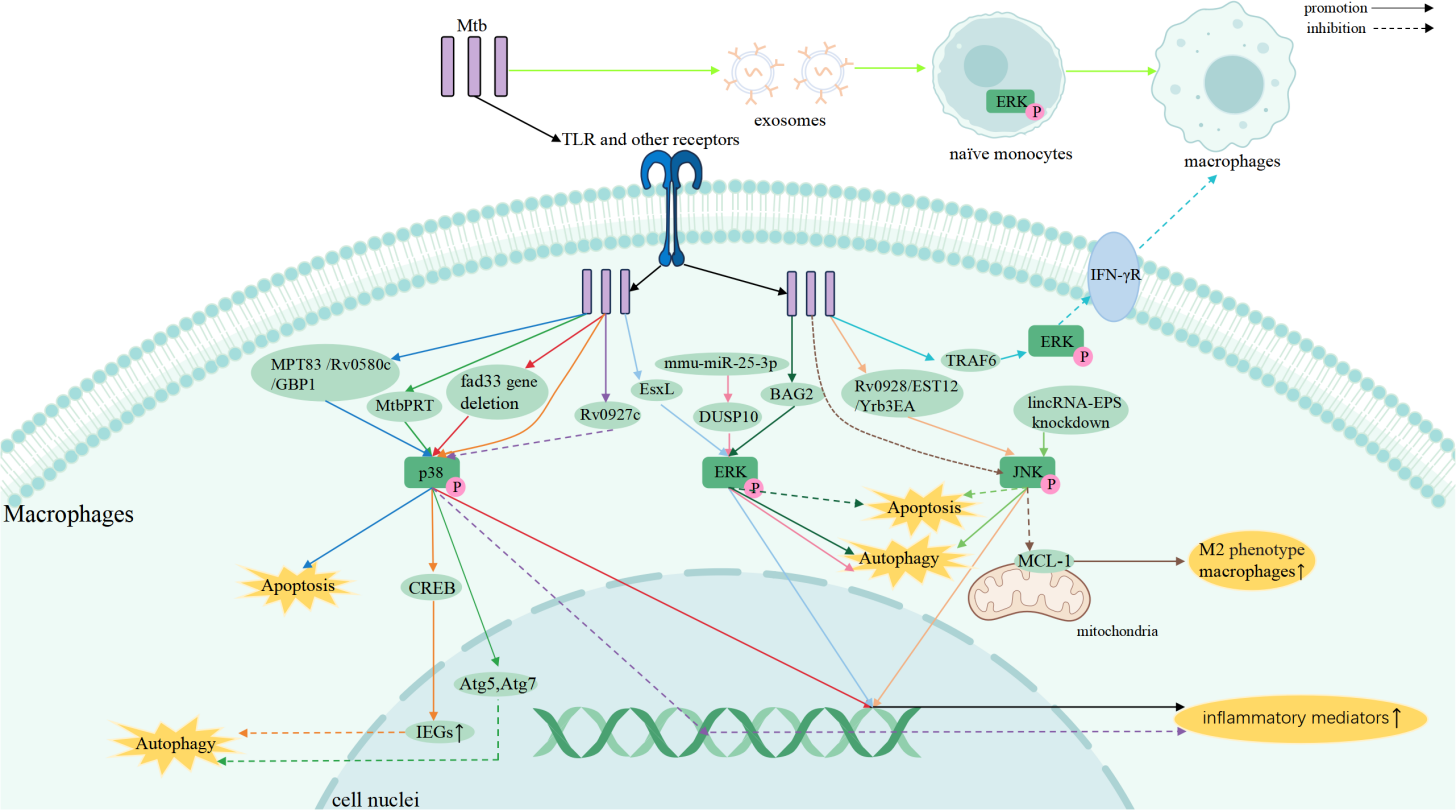
MAPK signaling in Mtb-infected macrophages coordinates inflammation, autophagy, and apoptosis through p38, ERK, and JNK pathways.

**Figure 3:**
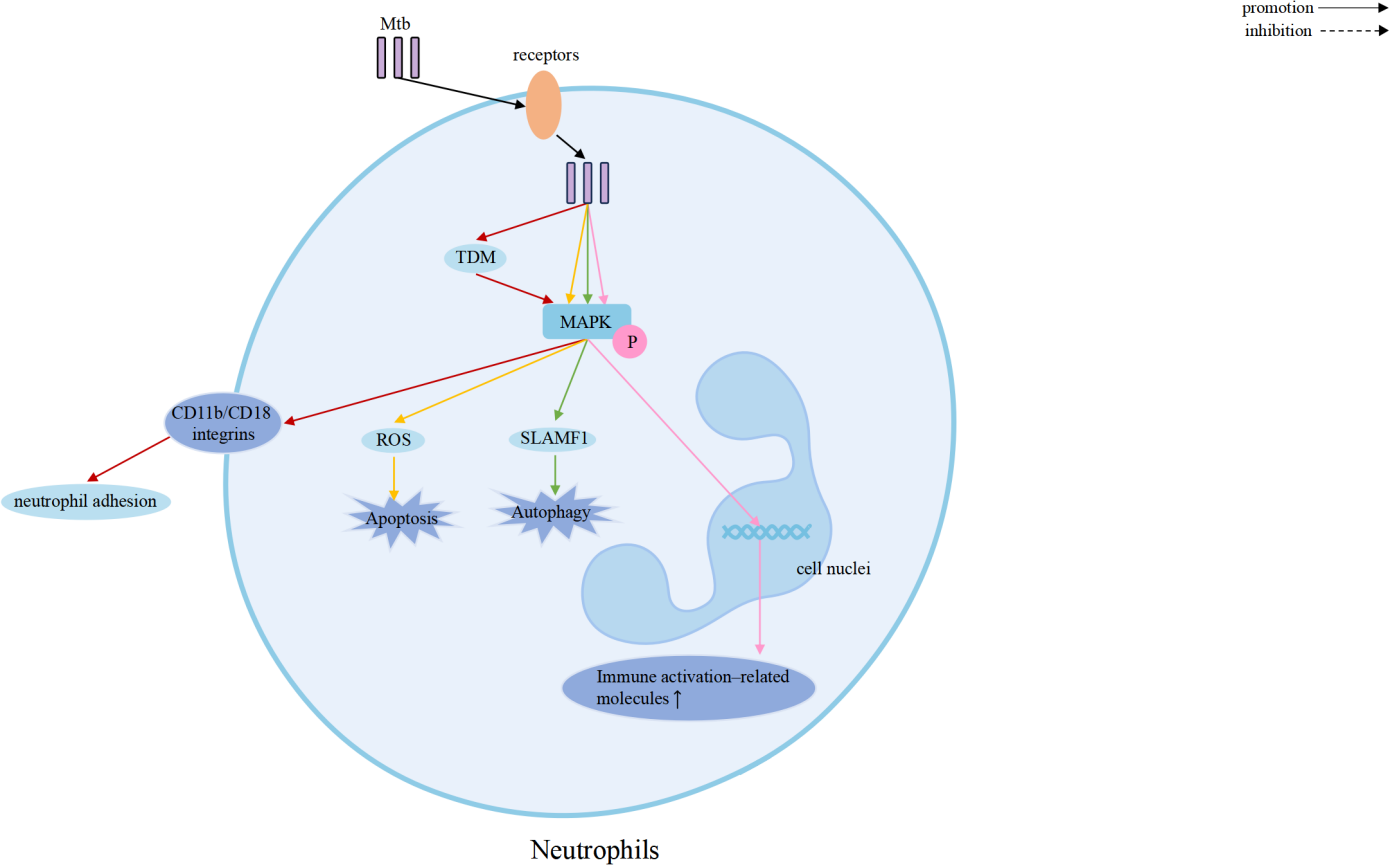
MAPK signaling in Mtb-infected neutrophils regulates inflammatory responses, antimicrobial activity, and cell fate decisions through coordinated activation of p38, ERK, and JNK.

**Figure 4:**
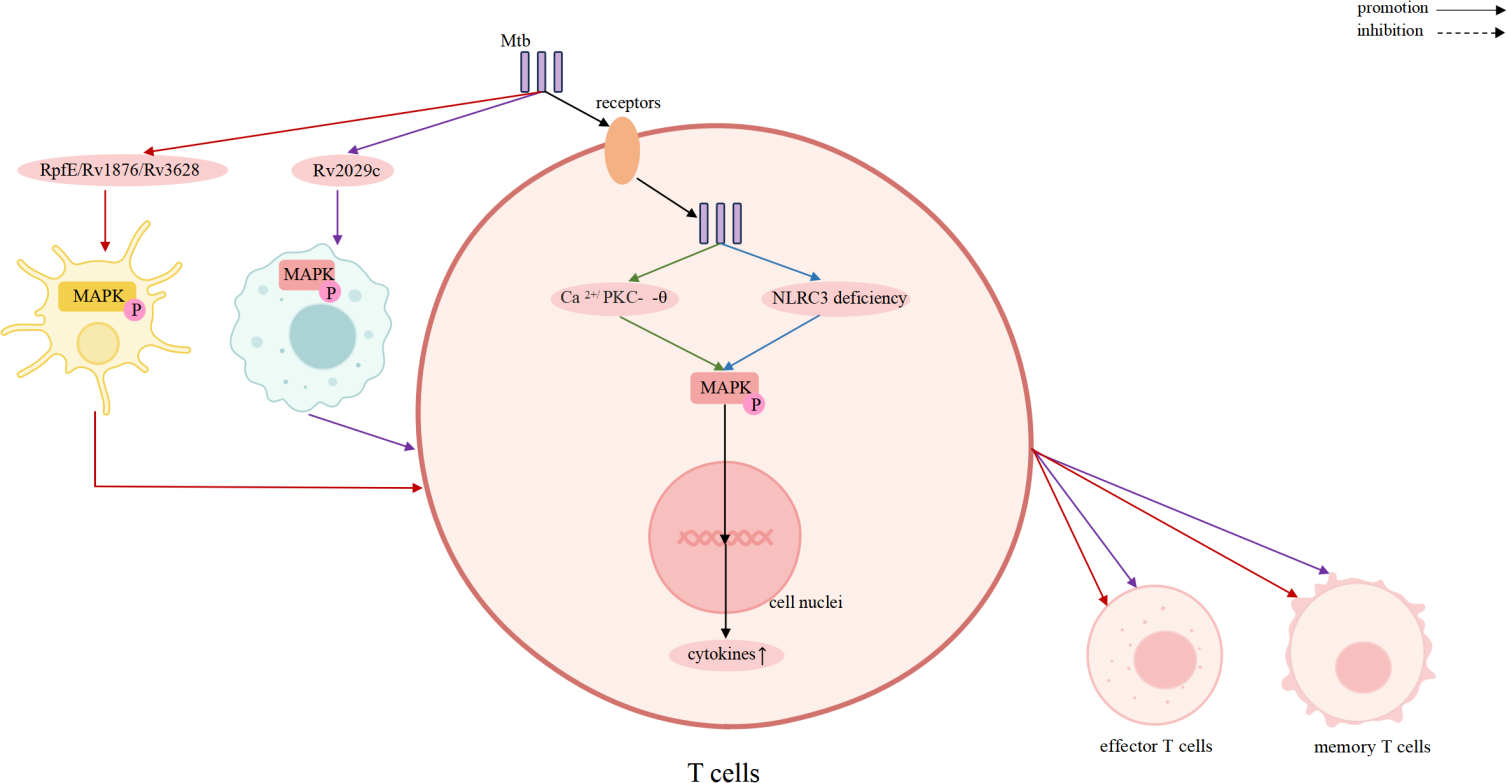
MAPK signaling in T cells drives effector/memory responses, linking innate activation to adaptive immunity against Mtb.

**Figure 5:**
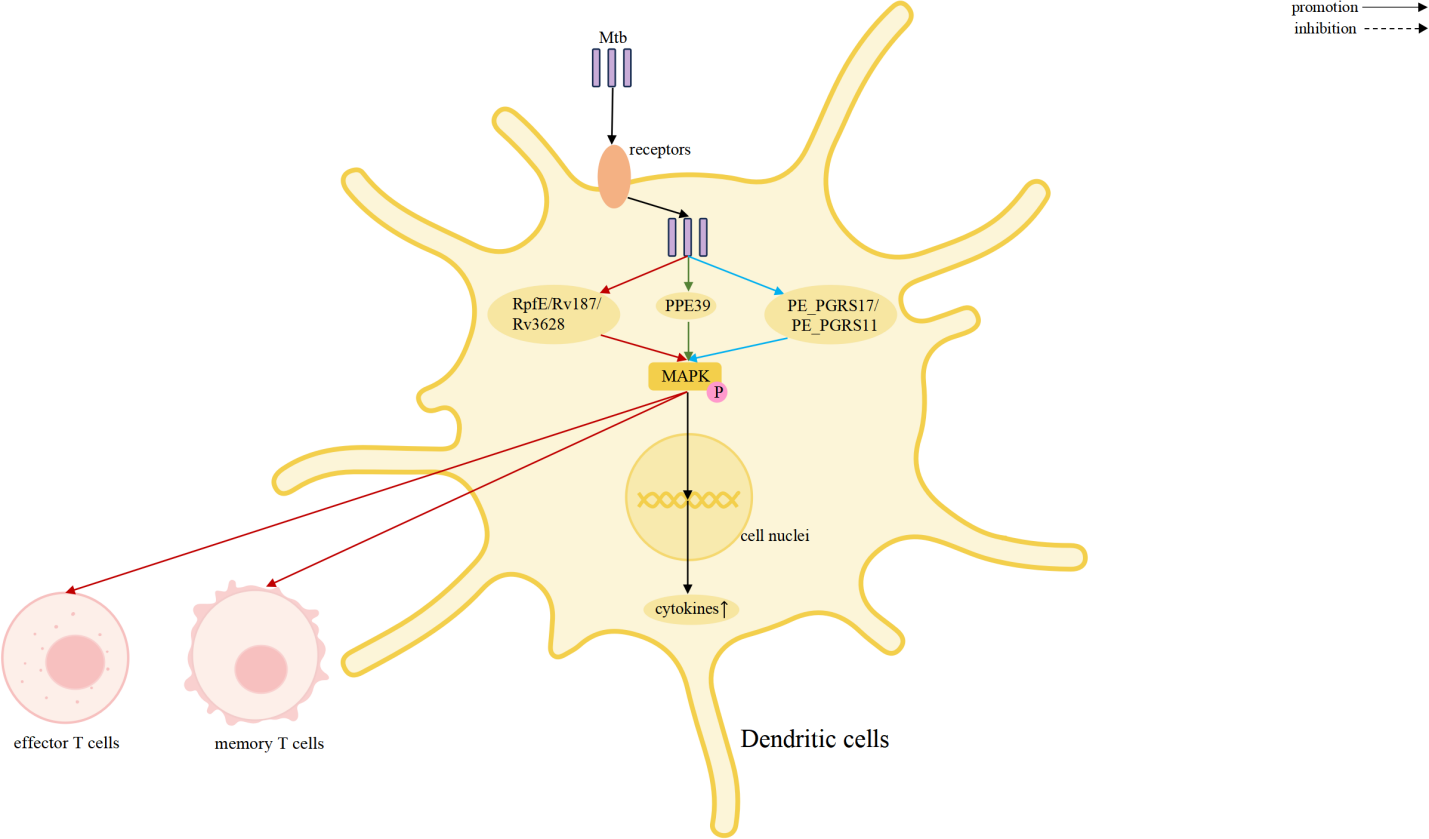
MAPK signaling in dendritic cells controls maturation, cytokine production, and T cell priming, integrating pattern recognition and Mtb-derived signals to shape adaptive responses.

The MAPK family plays a central role in regulating inflammation, stress responses, and antimicrobial defense, and it is closely associated with TB pathogenesis (15, 54, 55). Increasing evidence demonstrates that the three major MAPK pathways: p38, ERK, and JNK, shape host immunity by controlling cytokine production, autophagy, apoptosis, and cellular activation, thereby bridging innate and adaptive immunity (56, 57). Elucidating how MAPK participate in Mtb infection may provide new insights into host-directed therapeutic strategies. A schematic overview of MAPK-mediated regulation across immune cells is presented in **Figures 2**–**5**.

### 4.1 MAPK-dependent orchestration of inflammatory responses during Mtb infection

Inflammation is a hallmark of TB pathogenesis and a critical component of host defense against Mtb. MAPK signaling pathways play a pivotal role in orchestrating the magnitude, duration, and quality of inflammatory responses by regulating the production of pro-inflammatory cytokines across multiple immune cell populations (58).

Among the MAPK family, p38 MAPK plays a pivotal role in initiating and sustaining pro-inflammatory responses that contribute to early host defense (59). Multiple studies demonstrate that activation of p38 MAPK restricts intracellular Mtb survival in macrophages by driving the production of pro-inflammatory cytokines (60, 61). For example, deletion of the Mtb gene fadD33 leads to enhanced p38 activation, resulting in increased secretion of IL-1β, IL-6, and TNF-α, thereby limiting bacterial persistence within macrophages (62). Conversely, Mtb has evolved mechanisms to dampen this response; the virulence factor Rv0927c suppresses p38 and NF-κB activation, leading to reduced cytokine production and enhanced bacterial survival (63). The secreted effector Rv3722c targets the host adaptor TRAF3, simultaneously suppressing MAPK/NF-κB signaling and potentiating IFN-β production. This coordinated reprogramming shifts the immune response from an effective pro-inflammatory state toward an IFN-I-dominant environment, which can be detrimental to the host and favor Mtb persistence(M180). MAPK signaling intersects with the type I interferon (IFN-I) axis and the cGAS–STING pathway to coordinate macrophage responses during Mtb infection. Cytosolic Mtb DNA activates the cGAS–STING–TBK1 axis, inducing type I interferons and interferon-stimulated genes (ISGs). Simultaneously, Mtb engages TLR2 and TLR4, activating the MAPK pathway, particularly p38 MAPK, and NF-κB. This integration of MAPK, IFN-I, and cGAS–STING signaling enables macrophages to balance antimicrobial defense with the risk of immunopathology (64).

The ERK pathway also participates in inflammatory regulation (65, 66). ERK activation contributes to the amplification of pro-inflammatory responses, as demonstrated by Mtb EsxL–induced TNF-α production through coordinated activation of ERK and p38 signaling (33). However, ERK can also be hijacked by Mtb to dampen host immunity. Mtb-mediated redirection of TRAF6 signaling toward ERK1/2 or protein kinase C (PKC) leads to downregulation of the IFN-γ receptor on macrophages, thereby weakening macrophage activation and facilitating immune evasion (67). In addition, the Mtb protein Rv0309 suppresses inflammatory cytokine production through combined inhibition of NF-κB, ERK, and JNK pathways, further underscoring the immunomodulatory potential of ERK signaling (68). Beyond innate immune cells, MAPK signaling also modulates inflammatory responses in adaptive immunity. In tuberculous pleural effusion, Ca^2+^/PKC-θ–dependent activation of ERK1/2 and p38 enhances IL-2 and IFN-γ production by CD4^+^ T cells, strengthening host immune control over Mtb (69). Mtb PPE39,Mtb PE_PGRS17 and PE_PGRS11 proteins activate human dendritic cells via TLR2-dependent ERK1/2, p38 MAPK, and NF-κB signaling, promoting DC maturation, proinflammatory cytokine production, and enhanced CD4^+^ T cell activation (70, 71).

The JNK pathway primarily responds to cellular stress signals and contributes to inflammatory gene expression during Mtb infection (72, 73). Overexpression of Mtb Rv0928 enhances phosphorylation of JNK, p38, and NF-κB p65, resulting in elevated pro-inflammatory cytokine production in macrophages (74). Similarly, the Mtb EST12 protein activates the RACK1–JNK–AP-1–Myc axis, promoting transcription of IL-6, TNF-α, and iNOS, thereby enhancing nitric oxide production and bacterial clearance (75). In susceptible macrophages, persistent TNF signaling leads to sustained JNK activation, which synergizes with the canonical NF-κB/IRF1 axis to drive hyperinduction of IFN-β. This JNK-dependent amplification establishes a JNK/IFN-β/PKR circuit that triggers the integrated stress response, promoting macrophage dysfunction and granuloma necrosis (76).

Collectively, these studies illustrate that MAPK signaling pathways orchestrate inflammatory responses across innate and adaptive immune compartments, balancing antimicrobial defense with the risk of immunopathology during TB.

### 4.2 MAPK-mediated regulation of autophagy

Autophagy is a critical cell-intrinsic defense mechanism that limits intracellular Mtb survival, and MAPK signaling plays an essential role in regulating autophagic pathways during infection (77).

p38 MAPK contributes to autophagy regulation through both promotive and inhibitory mechanisms. Mtb phosphoribosyltransferase (MtbPRT) suppresses autophagy in an mTOR-independent manner by activating p38 MAPK and the EHMT2 methyltransferase, leading to histone hypermethylation at the Atg5 and Atg7 promoters and repression of autophagy-related gene expression (78). Activation of MAPK cascades, such as p38 and ERK1/2, often triggered by Mtb-secreted effector proteins (MoxR1) via pattern recognition receptors, intersects with the PI3K-AKT-mTOR pro-survival axis. This interaction leads to phosphorylation-dependent inhibition of ULK1, a key initiator of autophagy, suppressing autophagy and dampening pro-apoptotic signals like JNK, while enhancing anti-apoptotic pathways, thereby creating a cellular environment that favors Mtb survival (79).

Host-derived signals can engage MAPK signaling to enhance antimicrobial autophagy. In neutrophils, p38- and ERK-dependent upregulation of the co-stimulatory molecule SLAMF1 enhances Mtb-induced autophagy, further contributing to antimicrobial defense (80). Lysophosphatidylcholine (LPC) induces intracellular Ca^2+^ release and reactive oxygen species production via the cAMP–PKA–PI3K–p38 axis, promoting phagosome maturation and restricting Mtb growth (81). The ERK pathway is closely linked to autophagy initiation and progression. Inhibition of dual specificity phosphatase 10 (DUSP10) by mmu-miR-25-3p enhances ERK1/2 activation, accelerating autophagy initiation and reducing intracellular bacterial burden in BCG-infected macrophages (82). Additionally, ERK1/2-mediated phosphorylation of B-cell lymphoma 2 (BCL2) disrupts its interaction with Beclin-1 (BECN1), thereby activating autophagy while suppressing ER stress–induced apoptosis during Mtb infection (83).

### 4.3 Context-dependent roles of MAPK signaling in apoptosis

Apoptosis represents a double-edged sword in TB, as controlled cell death can restrict bacterial replication, whereas excessive or dysregulated apoptosis may exacerbate tissue damage. MAPK signaling pathways play context-dependent roles in determining cell fate during Mtb infection.

The p38 and JNK pathways are key mediators of apoptosis in infected macrophages. Mtb Rv0580c induces macrophage apoptosis through activation of the JNK/p38 axis, upregulating endoplasmic reticulum stress markers such as ATF-4, CHOP, and CHAC1, thereby limiting intracellular bacterial survival (84). Similarly, BCG-induced upregulation of guanylate-binding protein 1(GBP1) promotes macrophage apoptosis via phosphorylation of p38- and JNK-dependent targets, while GBP1 knockdown suppresses apoptosis and enhances bacterial persistence (85). In addition, the mycobacterial lipoprotein MPT83 induces macrophage apoptosis through the TLR2–p38–COX-2 pathway, suggesting a potential role for apoptosis-inducing antigens in TB vaccine development (86). JNK signaling also links oxidative stress to apoptotic pathways. Mtb Rv1654 activates the TLR4–ROS–JNK axis, triggering intrinsic apoptosis in macrophages (87). JNK signaling further integrates autophagy and cell survival pathways. Knockdown of the long intergenic noncoding RNA lincRNA-EPS activates JNK signaling, inducing autophagy and preventing apoptosis in BCG-infected macrophages (88). In patients with active TB, IL-17A produced by CD4^+^ T cells enhances macrophage autophagy via ERK1/2 activation, whereas IFN-γ induces autophagy through p38 signaling in both high- and low-responder patient groups, promoting intracellular Mtb clearance (89).

Certain Mtb strains induce excessive reactive oxygen species production through p38 activation, leading to heightened neutrophil apoptosis and potentially exacerbating inflammatory tissue damage (90).

### 4.4 MAPK-driven immune cell differentiation and shaping of adaptive immunity

In addition to regulating innate immune responses, MAPK signaling pathways play a crucial role in immune cell differentiation and the establishment of adaptive immunity during TB (91).

The ERK pathway is a key regulator of T cell activation and differentiation. NLRC3 deficiency enhances CD4^+^ T cell activation, proliferation, and cytokine production by relieving suppression of NF-κB and MEK–ERK signaling, thereby strengthening adaptive immune responses against Mtb (92). The Mtb RpfE protein binds to the TLR4 receptor on DCs, activating the p38/ERK MAPK and NF-κB signaling pathways. This activation promotes DCs maturation, which in turn induces the differentiation of naive CD4+ T cells into Th1 and Th17 lineages (93).

MAPK signaling also shapes the functional differentiation of macrophages, thereby influencing the long-term immunological landscape during Mtb infection. Inhibition of JNK signaling reduces Mcl-1 expression, driving macrophages toward an M2 phenotype characterized by anti-inflammatory activity and reduced tissue damage (94). Similarly, ERK1/2 activation suppresses IL-12 and iNOS expression, favoring M2 polarization in Mtb-infected macrophages (95). While M1 macrophages contribute to effective bacterial control, prolonged M1 polarization may lead to excessive inflammation, whereas M2 macrophages predominate in advanced tuberculous granulomas and are associated with tissue repair and immune regulation (94–96).

Through coordinated regulation of immune cell differentiation and polarization, MAPK pathways serve as critical bridges between innate and adaptive immunity, ultimately shaping the quality and durability of host responses to Mtb infection.

## 5 Drugs targeting the MAPK signaling pathway for the treatment of TB

Conventional anti-TB chemotherapeutics face significant challenges, including long treatment duration, adverse systemic toxicities, and the emergence of multidrug-resistant strains. In recent years, there has been an increase in research on drugs targeting the MAPK pathway, and more ideas have been provided for the clinical treatment of TB and improvement of the prognosis by searching for intervening targets in the process of immune response involved in the MAPK signaling pathway, and by developing biologically-targeted drugs. Recent studies have explored natural compounds, repurposed drugs, and so on that modulate p38, ERK, or JNK signaling to enhance host defense or limit immunopathology. The major drug candidates and their mechanisms are summarized below and in **Table 1**.

**Table 1:** Drugs that target the MAPK signaling pathway for treating TB.

Pathway	Agents (Effect)
p38	Pro-inflammatory: SQ109(induces M1 macrophage polarization) (98); Biapenem(increases cytokines) (99) Cell Death: Dexamethasone (inhibits necrosis) (100); JFD,SQ109(induce apoptosis) (97, 98)
ERK/p38	Enhancing Function: Vitamin B5 (promotes inflammatory responses, macrophage maturation, and Th1 and Th17 responses) (105) Suppressing Inflammation: Phloretin, Isorhamnetin(decrease multiple cytokines) (106, 107) Inducing Autophagy: IMQ (104)
JNK/p38	Pro-inflammatory: PBTZ169(increase pro-inflammatory cytokines) (108)
JNK	optimize inflammatory milieu: Allicin (Modulates cytokine production) (113) Anti-apoptotic: Curcumin (inhibits apoptosis) (111) Inducing Autophagy: Ajoene (112)
JNK/ERK/p38	Pro-inflammatory: Sudapyridine (WX-081) (increase pro-inflammatory cytokines) (109) Multi-pathway anti-inflammatory: Isoliquiritigenin(downregulates the expression of inflammatory mediators) (110)

### 5.1 Drugs targeting the p38 signaling pathway

Japoflavone D (JFD), a biflavonoid isolated from honeysuckle, has been investigated in Mtb-infected macrophage models in the preclinical stage. It directly targets Keap1 to inhibit Nrf2/SOD2 signaling, leading to ROS accumulation and p38-mediated apoptosis, thereby enhancing intracellular bacterial clearance (97). SQ109, a 1,2-ethylenediamine compound, is an anti-tuberculosis drug that directly targets MmpL3 and has completed phase I/II clinical trials. This study reveals for the first time that it induces M1 macrophage polarization and apoptosis through activation of the p38 MAPK pathway, thereby exerting immunomodulatory and protective effects in a mouse model of Mtb infection (98). Biapenem, a carbapenem antibiotic, has been shown to significantly enhance anti-tuberculosis immunity and reduce disease recurrence in a mouse model of Mtb infection by activating the p38 MAPK and promoting ROS production in macrophages. This study represents a preclinical mechanistic investigation of drug repurposing, not yet entered clinical trials for TB (99).

In addition to p38 activation–driven antimicrobial effects, several agents confer protective benefits by attenuating excessive p38-dependent inflammation. Glucocorticoids such as dexamethasone are already used clinically as adjunctive therapy for tuberculous meningitis. This study reveals for the first time that they prevent host cell necrotic death in Mtb-infected primary human macrophages and various cell models by activating the glucocorticoid receptor (GR), upregulating MKP-1, inducing p38 MAPK dephosphorylation, and stabilizing mitochondrial hexokinase II (100). Mtb ESAT-6 specifically inhibits IFN-γ production by directly activating the p38 MAPK signaling pathway in T cells. The p38 MAPK inhibitor SB203580 completely reverses this inhibitory effect in a dose-dependent manner, indicating that p38 MAPK is a critical node in the immunosuppressive function of ESAT-6 (101). Importantly, p38 inhibitors have been proposed as potential host-directed therapeutic agents in TB due to their ability to dampen excessive inflammation and prevent tissue destruction (102). However, given the essential role of p38 MAPK in antimicrobial responses, indiscriminate inhibition may impair macrophage activation, cytokine production, and intracellular bacterial clearance, potentially favoring pathogen persistence. Therefore, the therapeutic application of p38 inhibitors in TB requires careful consideration of timing, dosage, and disease stage to balance anti-inflammatory benefits against the risk of compromised host defense (103).

The p38 MAPK signaling pathway plays a dual role in TB infection. On one hand, agents such as JFD and SQ109 enhance bactericidal activity by activating p38 to promote ROS accumulation and macrophage apoptosis. On the other hand, excessive p38 activation leads to immunopathological damage, while glucocorticoids exert protective effects by dephosphorylating p38. Therefore, host-directed therapies targeting p38 must precisely balance its pro-inflammatory and anti-inflammatory effects, with careful consideration of the timing and dosage of intervention.

### 5.2 Drugs targeting the ERK signaling pathway

Imiquimod (IMQ), a TLR7 agonist, has been examined in Mtb-infected macrophage models, it activates the TLR7/MyD88 signaling pathway, initiating autophagy in the early phase through p38 MAPK/MEK-ERK1/2-mediated ROS generation (104). In a mouse model of Mtb H37Rv infection, vitamin B5 activates NF-κB, AKT, and p38 MAPK signaling pathways in macrophages, regulates ERK phosphorylation in a time-dependent manner, promotes inflammatory responses, macrophage maturation, and enhances Th1 and Th17 responses. This study represents a preclinical mechanistic investigation; although vitamin B5 is already used clinically as a nutritional supplement, it has not yet entered clinical trials for tuberculosis treatment (105). The anti-inflammatory activity of certain natural dietary compounds is mediated through their direct interaction with ERK and p38 MAPK. Phloretin and isorhamnetin downregulates the expression of inflammatory cytokines induced by IFN-γ or LPS by inhibiting the phosphorylation of p38 MAPK and ERK, and exhibits anti-inflammatory effects in a mouse model of Lipopolysaccharide-induced lung injury (106, 107).

### 5.3 Drugs targeting the JNK signaling pathway

JNK signaling represents another MAPK axis implicated in host-directed tuberculosis therapy. Several compounds modulating JNK have been evaluated in Mtb infection–related experimental settings.

PBTZ169, a benzothiazinone (BTZ) derivative, in macrophage infection models, enhances the intracellular bactericidal activity of macrophages against PBTZ169-resistant Mtb by promoting TAK1 phosphorylation, thereby activating NF-κB and MAPK (p38/JNK) signaling pathways and upregulating the expression of IL-6, IL-1β, TNF-α, IL-12, and type I interferons. This study is the first to reveal its immunomodulatory mechanism as a Phase II clinical trial candidate for TB treatment (108). Similarly, sudapyridine (WX-081), in macrophage infection models, activates NF-κB and MAPK signaling and upregulating the expression of pro-inflammatory cytokines. The study reveals its immunomodulatory mechanism as a Phase III clinical candidate for TB treatment (109).

Several plant-derived compounds have also been studied in Mtb-associated inflammatory or infection models. Isoliquiritigenin alleviates excessive inflammatory responses in a macrophage model of Mtb infection by inhibiting NLRP3 inflammasome activation, Notch1/NF-κB nuclear translocation, and MAPK (p38/ERK/JNK) phosphorylation, thereby downregulating the expression of inflammatory mediators (110). Curcumin has been shown in Mtb 19-kDa lipoprotein–stimulated macrophage models to selectively inhibit JNK activation, resulting in reduced apoptosis and inflammatory damage (111). Ajoene, a sulfur-containing compound derived from garlic, induces endoplasmic reticulum stress and autophagy by activating the IRE1α-JNK-ROS signaling axis, thereby significantly inhibiting the intracellular survival of Mtb H37Rv in a macrophage model (112). These studies are *in vitro* mechanistic investigations and have not yet progressed to animal models or clinical trials for TB. In a mouse model infected with Mtb H37Rv as well as MDR/XDR strains, Allicin selectively activates SAPK/JNK while inhibiting p38 MAPK in macrophages, leading to a moderate increase in IL-1β/IL-12 and a decrease in TNF-α/IL-10, thereby optimizing the inflammatory milieu and enhancing anti-tuberculosis immune responses (113).

## 6 Discussion

To summarize, the p38, ERK, and JNK signaling pathways constitute central regulatory axes that orchestrate key cellular processes, including inflammation, autophagy, apoptosis, and immune cell differentiation during Mtb infection. Rather than functioning as isolated linear cascades, these MAPK pathways exhibit extensive crosstalk and context-dependent integration, enabling immune cells to dynamically balance antimicrobial defense and tissue homeostasis. Accumulating evidence indicates that MAPK signaling exerts a fundamentally bidirectional influence on TB immunity. On one hand, MAPK activation promotes protective host responses, such as the production of pro-inflammatory cytokines (TNF-α, IL-1β, and IL-6), induction of autophagy and apoptosis in infected macrophages and neutrophils, and the differentiation and activation of effector T cells, collectively contributing to the restriction of intracellular Mtb growth and reduction of pulmonary bacterial burden. On the other hand, excessive or dysregulated MAPK activation can amplify inflammatory cascades, drive pathological tissue remodeling, and exacerbate lung injury, fibrosis, and cavitation. These opposing outcomes underscore MAPK signaling as a finely tuned immunological rheostat in TB pathogenesis rather than a unidirectional antimicrobial pathway.

MAPK signaling during Mtb infection represents a dynamic host–pathogen interface rather than a unidirectional antimicrobial pathway. Multiple Mtb-derived proteins—including Rv1876, Rv2029c, RpfE, Rv3628, PPE39, PE_PGRS17, and PE_PGRS11—activate ERK, p38, and JNK–dependent mechanisms, promoting dendritic cell maturation, Th1/Th17 polarization, and pro-inflammatory cytokine production. Similarly, EST12 and EsxL enhance antimicrobial responses through coordinated ERK/p38 or RACK1–JNK–AP-1–Myc activation, reinforcing nitric oxide–mediated bacterial clearance. In contrast, Mtb employs dedicated virulence factors to dampen or reprogram MAPK outputs. Rv0927c and Rv0309 suppress p38, ERK, JNK, and NF-κB signaling to reduce inflammatory cytokine production, whereas Rv3722c targets TRAF3 to attenuate MAPK/NF-κB activation while promoting type I interferon responses, thereby shifting immunity toward a persistence-favoring state. Beyond cytokine regulation, MAPK pathways are exploited to control cell fate and autophagy. Rv0580c and Rv1654 activate JNK/p38-driven apoptosis through ER stress and ROS signaling, while MoxR1-mediated crosstalk with the PI3K–AKT–mTOR axis inhibits ULK1-dependent autophagy and suppresses pro-apoptotic signaling. In addition, MtbPRT activates p38–EHMT2 signaling to epigenetically repress Atg5 and Atg7 transcription, further restricting autophagic clearance.

Moreover, MAPK signaling serves as a central integrative node during Mtb infection, linking innate sensing to adaptive immune polarization and metabolic regulation. Through TLR-dependent activation, ERK, p38, and JNK pathways drive cytokine production that promotes Th1 and Th17 differentiation, bridging innate and protective T cell responses. Concurrently, MAPK signaling intersects with the type I interferon axis, mTOR-mediated autophagy control, and the cGAS–STING pathway, collectively determining the balance between antimicrobial defense and immunopathology. Dysregulation of this network may favor bacterial persistence and tissue damage. While direct evidence connecting MAPK signaling to foamy macrophage formation is limited—pharmacological inhibition of ERK, p38, or JNK does not suppress Mtb-induced lipid droplet accumulation (114)—macrophage metabolic states strongly dictate foam cell differentiation and bacterial control. Glycolytic macrophages favor apoptosis and pathogen clearance, whereas fatty acid oxidation–dependent subsets resist foam cell formation but allow bacterial replication (115, 116). And strain-specific differences further modulate outcomes: virulent strains inhibit apoptosis and promote necrosis, whereas attenuated strains induce apoptosis more effectively (26). These findings highlight how MAPK signaling, metabolic state, and bacterial virulence factors intersect to shape macrophage fate, antimicrobial responses, and granuloma dynamics, providing a cohesive framework for understanding host–pathogen interactions and guiding MAPK-targeted host-directed therapies in TB.

Existing anti-tuberculosis drugs have limitations, including suboptimal efficacy, prolonged treatment durations, and the emergence of diverse mechanisms of drug resistance, which have rendered many current therapeutic agents less effective than anticipated, particularly in the context of multidrug-resistant tuberculosis (MDR-TB) and extensively drug-resistant tuberculosis (XDR-TB). The high global burden of MDR-TB, coupled with the limited availability of effective treatment options, underscores the urgent need for the development of novel targeted drugs. Advances in understanding the molecular mechanisms of Mtb pathogenesis and resistance, combined with innovative drug discovery approaches such as structure-based drug design, high-throughput screening, and repurposing of existing drugs, are critical to addressing these gaps.

This review provides an overview of the developments in the study of how different medications affect the MAPK pathway and engage TB. Pharmacological interventions targeting the MAPK pathway offer unique advantages, including the potential to regulate inflammatory responses and promote bacterial clearance. However, despite encouraging preclinical results, the translation of MAPK-targeted therapies into clinical practice remains limited. Differences between human and animal MAPK signaling, context-dependent effects of pathway modulation, and the risk of systemic toxicity present major challenges. Further studies are required to achieve selective, tissue-specific modulation of MAPK pathways to minimize off-target effects.

Given the extensive involvement of the MAPK pathway in cellular functions, developing targeted therapies with localized effects while minimizing systemic side effects remains a significant challenge. Future research should focus on refining MAPK-targeted interventions through precision medicine approaches, incorporating host-directed therapies, and optimizing combination treatment strategies. Personalized treatment plans based on MAPK pathway modulation may provide novel avenues for MDR-TB management, potentially integrating immunomodulatory agents to enhance therapeutic efficacy and reduce drug resistance. Clarifying the relationship between the MAPK signaling and TB pathogenesis could pave the way for innovative treatment strategies, ultimately improving clinical outcomes in drug-resistant TB cases.
